# Depth-Dependent Changes in Collagen Organization in the Human Peripapillary Sclera

**DOI:** 10.1371/journal.pone.0118648

**Published:** 2015-02-25

**Authors:** Jacek K. Pijanka, Martin T. Spang, Thomas Sorensen, Jun Liu, Thao D. Nguyen, Harry A. Quigley, Craig Boote

**Affiliations:** 1 Structural Biophysics Group, School of Optometry and Vision Sciences, Cardiff University, United Kingdom; 2 Diamond Light Source, Didcot, United Kingdom; 3 Department of Biomedical Engineering, The Ohio State University, Columbus, Ohio, United States of America; 4 Department of Mechanical Engineering, Johns Hopkins University, Baltimore, Maryland, United States of America; 5 Glaucoma Center of Excellence, Wilmer Ophthalmological Institute, Johns Hopkins University School of Medicine, Baltimore, Maryland, United States of America; University of Reading, UNITED KINGDOM

## Abstract

**Purpose:**

The collagen structure of the human peripapillary sclera plays a significant role in determining optic nerve head (ONH) biomechanics, and is therefore of interest in the study of glaucoma. The aim of the current work was to map the anisotropic collagen structure of the normal human peripapillary sclera as a function of tissue depth.

**Methods:**

Wide-angle x-ray scattering was used to quantify collagen fibril orientation at 0.5mm intervals across six 150μm-thick serial sections through the peripapillary sclera of eight normal European-derived human eyes. Two structural parameters were measured: 1) the relative number of fibrils preferentially aligned at a given angle within the tissue plane, 2) the degree of collagen alignment (anisotropy).

**Results:**

The inner-most one-third of the peripapillary scleral stroma (nearest to the choroid) was characterised by collagen fibrils either randomly arranged or preferentially aligned radially with respect to the ONH. In contrast, the outer two-thirds of the tissue was dominated by a circumferential arrangement of collagen encircling the ONH. In all tissue regions the degree of collagen anisotropy peaked in the mid-stroma and progressively decreased towards the tissue surfaces, with the largest depth variations occurring in the inferior-nasal quadrant, and the smallest occurring in the superior-nasal quadrant.

**Conclusions:**

Significant, region-specific variations in collagen structure are present in the human peripapillary sclera as a function of depth. In normal eyes, the circumferential collagen fibril architecture is most prominent in the outer two-thirds of the stroma, possibly as a mechanical adaption to more effectively support the lamina cribrosa at the level of its insertion into the scleral canal wall.

## Introduction

The sclera is the white, fibrous tissue that forms 85% of the ocular tunic in humans[1]. The material properties of the sclera are paramount to its function as the eye's main load bearing tissue. Together with the cornea, the sclera must be precisely shaped in order to cooperatively focus an image on the retina, while also providing important mechanical support for the vulnerable optic nerve axons as they exit the eye close to the posterior pole[2].

Scleral material properties are heavily influenced by the tissue microstructure. The scleral stroma constitutes the bulk of the tissue thickness and comprises a layered scaffold of collagen fibrils (99% type I) embedded in an interfibrillar matrix of water, non-fibrous collagens, elastic fibers and proteoglycans, and populated by fibroblast cells[2]. Collagen molecules form fibrils that are assembled into bundles and these into stacked lamellae that lie roughly parallel to the tissue surface, although they are increasingly interwoven in the deeper tissue[3]. The orientation of the collagen fibrils in sclera is highly dependent on region and appears mechanically adapted to withstand wall tension derived from the intraocular pressure (IOP) and pull of the extraocular muscles[4, 5]. Of particular note, the peripapillary sclera bordering the optic nerve head (ONH) is dominated by circumferentially oriented collagen[4–8] and elastin[9, 10] which has been suggested to serve a neuroprotective function by limiting scleral canal expansion under fluctuating IOP[11–13]. Thus, detailed quantitative information on peripapillary scleral collagen architecture will benefit efforts to understand and model the tissue’s physiological load-bearing behaviour and its potential clinical implications, in particular for glaucoma.

A number of techniques have been developed to quantify collagen fibril orientation in the sclera, including second harmonic generation (SHG) non-linear microscopy[7, 8, 14], small-angle light scattering (SALS)[14–16] and wide-angle x-ray scattering (WAXS)[5, 7]. Previously we have applied WAXS to obtain quantitative maps of collagen orientation in the human peripapillary sclera[7]. However these data are limited to thickness-averaged measurements, while depth-resolved information in this region of the sclera is lacking. Previous work by our group[7] and others[15] has suggested that scleral collagen fibril architecture changes significantly as a function of depth; however, these studies have been so far restricted to localised regions of the peripapillary[7] and mid-posterior[15] sclera. The purpose of the current work was to obtain detailed, quantitative maps of collagen orientation in the human peripapillary sclera at multiple levels through the stromal thickness, and to use these to determine how the anisotropic collagen structure of the tissue varies as a function of depth.

## Methods

### Ethical information

This research was approved by the Human Science Ethical Committee (School of Optometry and Vision Sciences, Cardiff University, UK) and the South East Wales Research Ethics Committee (Cardiff, UK). The institutional review board approved the use of all tissue described in the study and a waiver of consent was given for the donor scleras. All tissue was obtained in accordance with the tenets of the Declaration of Helsinki, and local ethical rules were adhered to throughout. All experimental procedures were performed in accordance with the Declaration of Helsinki.

### Tissue details and specimen preparation

**Fig 1:**
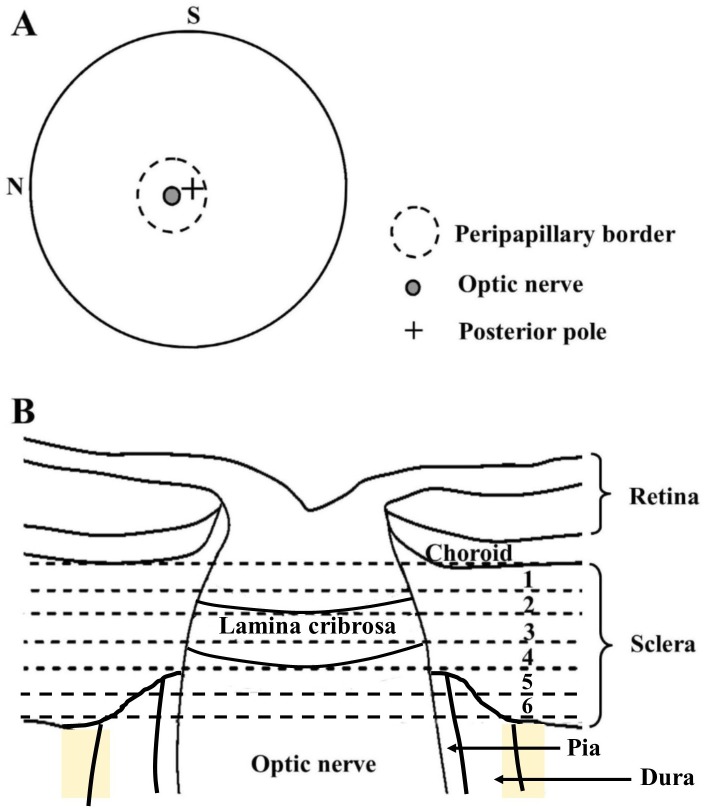
Data sampling locations. A) Location of peripapillary region of interest on posterior view of a right human eye. The superior (S) and nasal (N) directions are marked. B) Schematic cross-section through peripapillary sclera and ONH of human eye. Locations of the six serial 150μm-thick sections taken for WAXS analysis are numbered and bounded by dashed lines.

Eight human eyes from seven European-derived donors, were obtained from the National Disease Research Interchange (NDRI) within 48 hours post-mortem. None of the eyes had any documented history of scleral disease, inflammatory conditions or surgery. The mean age of the donors was 68 ±10 yrs. The eyes were shipped on dry ice and preserved in balanced salt solution (BSS, Alcon, Inc., Fort Worth, TX). The surrounding fat, muscle and episcleral tissues were gently removed from each eye, and the optic nerve excised with a razor blade flush to the sclera. The cornea and anterior sclera were then excised and the lens, retina and choroid removed. The intact posterior scleras were removed to 4% paraformaldehyde (Electron Microscopy Sciences, Hatfield, PA) and stored at 4°C until the time of x-ray experiments. Our previous work has shown that this mild fixation method does not affect WAXS collagen orientation measurements[17, 18]. To allow depth-profiling of the peripapillary sclera full-thickness circular specimens, centered on the ONH, were excised from each fixed scleral specimen using a 6mm diameter biopsy punch (Fig. 1A), and the resulting tissue pieces separated into six 150μm thick serial sections (Fig. 1B) using a sledge cryo-microtome (HM440E, Microm, Walldorf, Germany). After cutting, the sections were immediately returned to the paraformaldehyde fixative prior to transportation to the Diamond Light Source (Didcot, UK) for subsequent WAXS experiments.

### WAXS data collection

Diamond macromolecular crystallography beamline I02 was used to record WAXS patterns across each specimen at 0.5mm (horizontal) × 0.5mm (vertical) intervals, using an x-ray beam of wavelength 0.098nm and a cross-sectional diameter of 0.1mm. For examination, specimens were wrapped in polyvinylidene chloride film to prevent tissue dehydration, and mounted inside Perspex (Lucite Group Ltd, Southampton, UK) chambers with Mylar (DuPont-Teijin, Middlesbrough, UK) windows. The incident x-ray beam was directed perpendicular to the surface of each flattened specimen. Initial specimen alignment was achieved via an in-line microscope directed along the incident x-ray beam direction. WAXS patterns, each resulting from an x-ray exposure of 3 to 5s were recorded electronically on a Pilatus-6MF silicon pixel detector (Dectris Ltd, Baden, Switzerland) placed 350mm behind the specimen position. Specimen translation between exposures was achieved using an integrated x-y motor stage.

### WAXS data processing

As described elsewhere[5, 7], analysis of the ~1.6nm intermolecular collagen signal in the scleral WAXS pattern can provide a quantitative measure of bulk collagen fibril orientation, as an average value within the tissue volume sampled by the x-ray beam. For every sampled location across each specimen, we obtained two measurements: 1) the relative number of fibrils preferentially aligned at a given angle within the tissue plane (over and above the population of fibrils that are arranged isotropically), 2) the degree of collagen anisotropy (amount of preferentially aligned collagen as a proportion of the total fibrillar collagen content).

**Fig 2:**
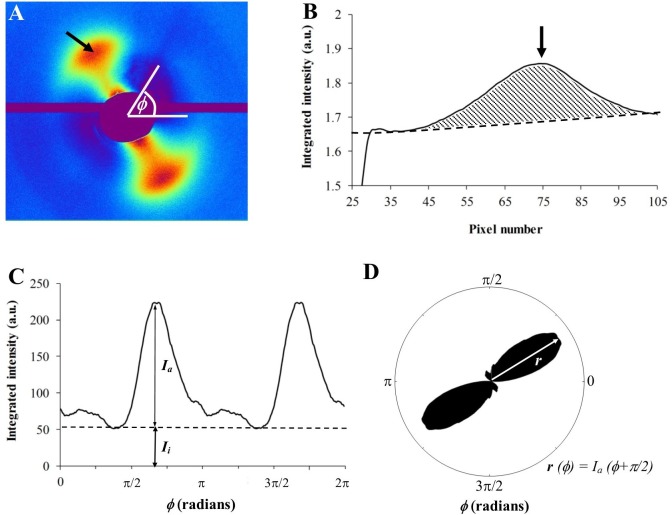
X-ray scattering data analysis. A) WAXS pattern from peripapillary human sclera. The spread of x-ray scatter intensity as a function of azimuth angle, φ, around the collagen intermolecular reflection (arrow) contains information about the orientation distribution of fibrils. B) Power-law background function (broken line) fitted to a radial profile (solid line) through pattern shown in A. For each pattern, independent background functions were fitted and subtracted along 256 equally spaced radial directions (every 1.4°), enabling the collagen signal to be isolated and extracted in two dimensions. *Arrow:* Collagen peak. C) Angular x-ray scatter intensity profile of pattern shown in A. The scatter intensity may be separated into that arising from isotropic collagen, Ii, and that arising from preferentially aligned fibrils, Ia. D) Aligned collagen scatter displayed in a polar coordinate system. The plot shape reveals the collagen anisotropy. The length of vector, r(φ), is proportional to the relative number of fibrils preferentially aligned at angle, φ+π/2.

For each raw WAXS pattern (Fig. 2A), a bespoke two-dimensional background fitting algorithm was used to fit and remove scatter from the specimen cell and non-collagen scleral components[19]. Briefly, 256 radial profiles from pattern center to beyond the collagen signal were generated and a unique fitted power-law background function independently subtracted from each (Fig. 2B). The isolated collagen scatter peak for each of the 256 angular directions (intervals of 1.4°) was then normalised against fluctuations in x-ray beam current and exposure time, radially integrated, and the resulting values extracted to angular bins, using a combination of Optimas 6.5 (Media Cybernetics Inc., Marlow, UK) image analysis software and Excel (Microsoft, Reading, UK). The resulting profiles were then each divided into isotropic and anisotropic scatter components (Fig. 2C) and the latter plotted in a polar vector coordinate system using Statistica 7 (StatSoft Ltd, Bedford, UK). A π/2 angular shift was applied to account for equatorial scatter. Every sampled point could then be represented by a polar vector plot, in which the vector length gave the relative number of fibrils preferentially aligned in the vector direction (Fig. 2D). Individual plots were assimilated using Excel into montages showing the direction and associated angular distribution of preferential fibril orientations across each specimen.

Spatial distribution maps of collagen anisotropy were produced in MATLAB software (The MathWorks Inc., Natick, MA) by dividing the integrated value of the aligned scatter distribution by the corresponding integrated value of the total scatter distribution, according to Eq. 1, as described previously[7].

$$Anisotropy=\frac{\int_{0}^{2\pi }I_{a}d\varphi }{\int_{0}^{2\pi }(I_{a}+I_{i})d\varphi }$$ (1)

where *I*_*a*_ and *I*_*i*_ represent the preferentially aligned and isotropic components of the collagen scatter at each angle, φ.

### Statistical analysis

**Fig 3:**
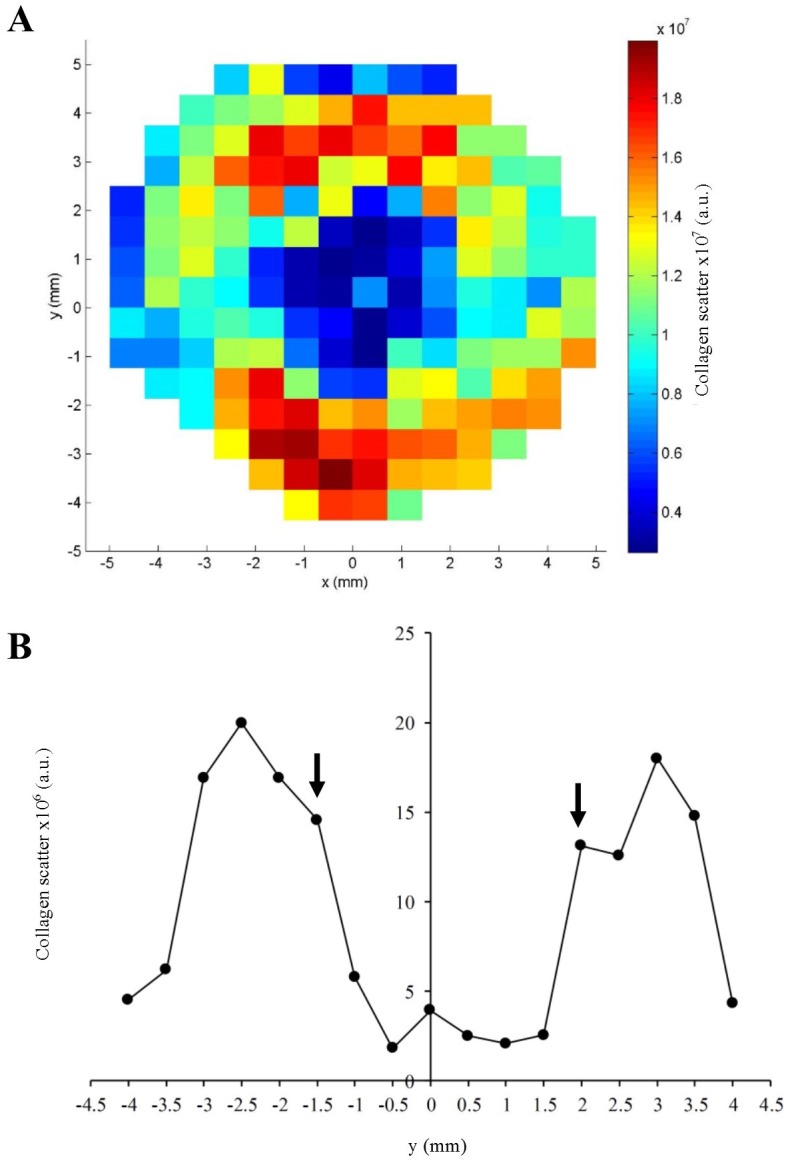
Location of scleral canal using x-ray data. A) Map of total collagen x-ray scatter across a mid-stromal section of the human peripapillary sclera and ONH. Smaller scatter values, indicative of lower collagen density, typify the central, porous lamina cribrosa. B) Vertical data transect through the map shown in A). The abrupt reduction in collagen scatter when passing from the peripapillary sclera into the lamina may be used to locate the scleral canal edge (*arrows*).

For statistical evaluation of fibril alignment as a function of tissue depth, the anisotropy maps were divided into four quadrants: superior-nasal, superior-temporal, inferior-nasal and inferior-temporal. This was done independently for each section, using the abrupt drop-out in total collagen scatter values at the scleral canal edge to detect and remove the much weaker collagen scatter data from the highly porous lamina region (visible in the raw data from the mid/outer sections) (Fig. 3). In this way we were able to account for the well-characterised[20–22] variation in scleral canal diameter resulting from the tapering of the optic nerve (Fig. 1B) and also account for any variation in canal shape and size between between donors. For each region, the mean and standard deviation were calculated and the pooled anisotropy values compared between section numbers (and hence depths) using two-tailed t-tests in MATLAB. To account for lower variability between eyes from the same donor, corresponding values for the fellow pair were firstly averaged for subsequent statistical analysis. For each specimen an average of 43 unique measurements of collagen anisotropy were recorded from each peripapillary scleral quadrant, each from a discrete location within the specimen. This generated an average sample size of 301 for pooled t-test comparisons of inter-section anisotropy. To verify that this was a sufficient sample size, we performed an a-priori power calculation in Statistica 7, which indicated that n = 301 is sufficient to detect a medium anticipated effect size (Cohen’s d = 0.5) at a statistical power level of 0.8 and a probability level of 0.001.

## Results

**Fig 4:**
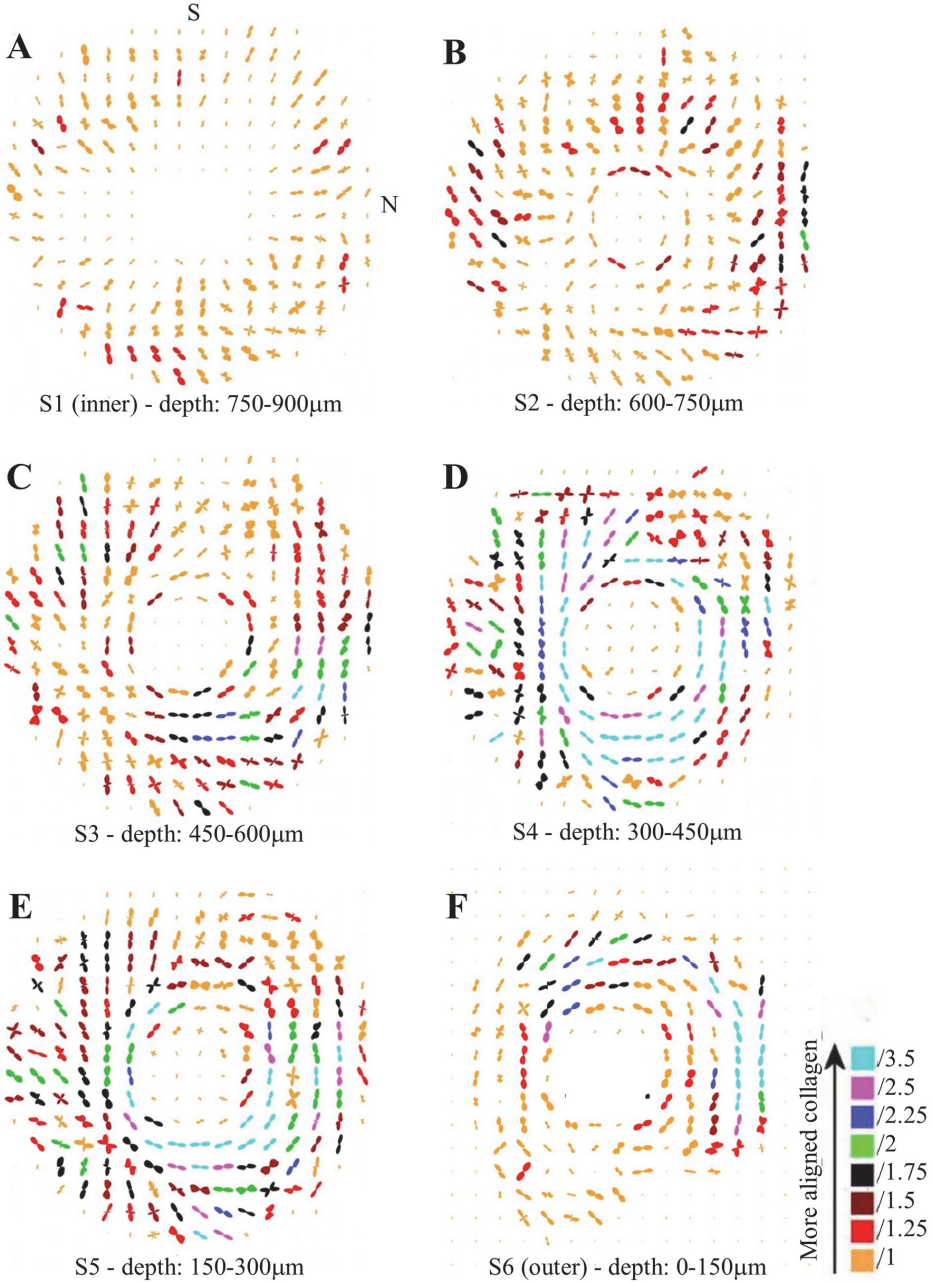
Collagen orientation maps. Representative polar vector maps of preferential collagen fibril orientation across six serial sections (S1-S6) of human peripapillary sclera, at the stromal depths indicated. The inner section refers to that bordering the choroid, whereas the outer refers to that adjacent to the episclera. The plots have been scaled according to the color key and the superior (S) and nasal (N) aspects are indicated. Sampling interval: 0.5mm.

Fig. 4 shows representative polar vector maps of preferred collagen fibril orientation in six serial sections through the human peripapillary sclera. In the innermost 150μm of stroma, adjacent to the choroid (Fig. 4A), the shape of the individual vector plots indicated that the collagen was preferentially oriented in a radial direction. In the next section (Fig. 4B), the first signs of tangential collagen were evident at the edge of the scleral canal, while the remainder of the tissue peripheral to this was characterised by either radial or random fibril alignment. Thereafter, the tissue was dominated by circumferential collagen which encircled the ONH (Fig. 4C-F). The amount of aligned collagen forming the circumferential structure varied markedly with anatomical position, as indicated by the color coding of the plots.

**Fig 5:**
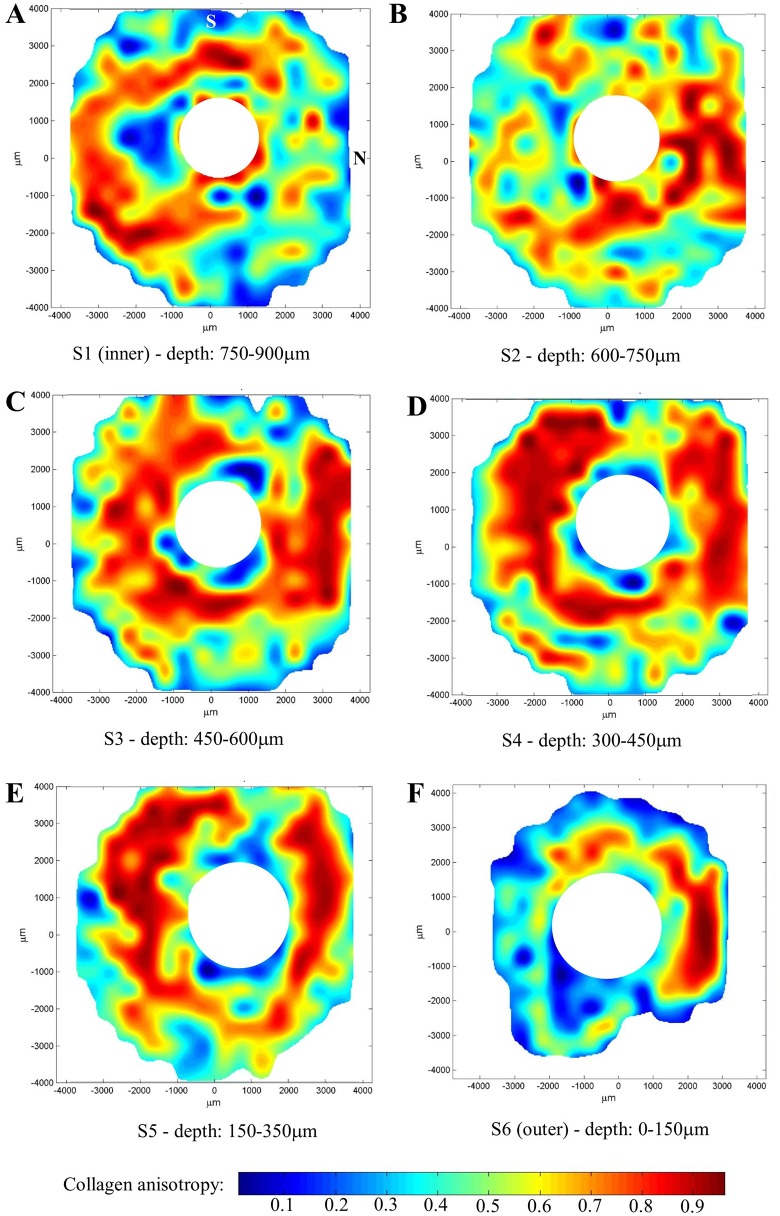
Collagen anisotropy maps. Representative contour maps showing degree of collagen anisotropy across six serial sections (S1-S6) of human peripapillary sclera, at the stromal depths indicated. The inner section refers to that bordering the choroid, whereas the outer refers to that adjacent to the episclera. The superior (S) and nasal (N) aspects are indicated. Data from the lamina cribrosa region has been removed for clarity.

As the size of the polar vector plots are affected by variations in tissue thickness, hydration and collagen volume fraction[19], a more accurate measure of the degree of collagen alignment (anisotropy) may be obtained by dividing the preferentially aligned collagen scatter by the total scatter from all fibrillar collagen (Fig. 2C and Eq. 1). The resulting values are presented as anisotropy contour maps in Fig. 5. Large spatial variations in the degree of collagen alignment were evident in the two inner-most layers of the scleral stroma (Fig. 5A,B). In traversing the next one-third of the stroma from an intraocular to extraocular direction (Fig. 5C,D), corresponding to the appearance of the circumferential collagen orientation (Fig. 4C,D), the tissue displayed progressively higher anisotropy, and at a depth of 300–450μm from the outer surface the fibrils formed an almost continuous "ring" of high anisotropy that circumscribed the ONH (Fig. 5D). The exception was the superior-nasal region which retained markedly lower anisotropy (Fig. 5C,D). In the outer one-third of the stroma, the anisotropy progressively decreased again (Fig. 5E,F).

**Fig 6:**
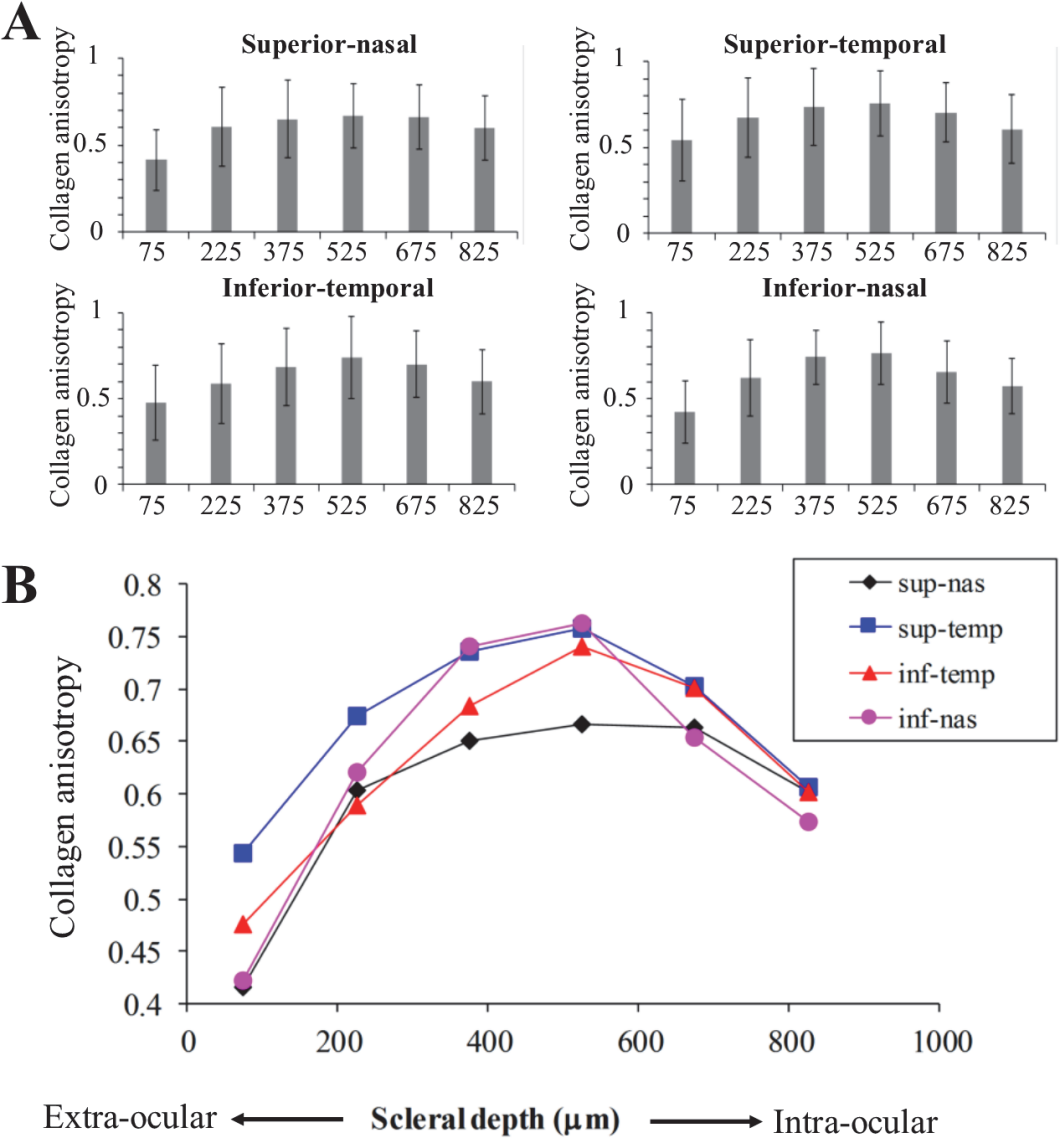
Anisotropy depth-profiles. Mean collagen anisotropy vs depth (mid-section), by peripapillary region, averaged over eight human scleras. A) Bar charts to show trend in separate regions, where bar height represents mean and error bars denote standard deviation. B) Composite graph showing trend in mean for all regions.

Mean collagen anisotropy for each peripapillary quadrant, averaged over eight human eyes, is plotted as a function of depth in Fig. 6. These results confirmed that the collagen anisotropy was highest in the mid-stroma. At a depth of 450–600μm from the extraocular scleral surface anisotropy peaked at an average of 0.72, indicating that at this depth 72% of collagen fibrils were preferentially arranged circumferentially, with the remaining 28% being randomly arranged. From this depth, anisotropy progressively decreased towards the intraocular and extraocular surfaces and, further, the manner of variation with depth was region-specific. The inferior-nasal quadrant demonstrated the largest variations in collagen anisotropy with depth, whereas the superior-nasal showed the smallest variation. This trend was confirmed by a systematic, statistical comparison of mean anisotropy between sections (Tables 1–4), which indicated significant differences in 12 out of 15 tests for the inferior-nasal region (Table 4), as compared to only 7 out of 15 for the superior-nasal (Table 1).

**Table 1:** Statistical comparison of mean collagen anisotropy at different depths for the superior-nasal peripapillary scleral quadrant (two-tailed t-tests).

Section (mean depth, μm)	S1 (825)	S2 (675)	S3 (525)	S4 (375)	S5 (225)	S6 (75)
S1(825)			*			***
S2(675)						***
S3(525)					*	***
S4(375)						***
S5(225)						***
S6(75)						

Significance is indicated at three probability levels

* p < 0.05

** p < 0.01

*** p < 0.001.

**Table 2:** Statistical comparison of mean collagen anisotropy at different depths for the superior-temporal peripapillary scleral quadrant (two-tailed t-tests).

Section (mean depth, μm)	S1 (825)	S2 (675)	S3 (525)	S4 (375)	S5 (225)	S6 (75)
S1(825)		**	***	***	*	
S2(675)			*			***
S3(525)					**	***
S4(375)						***
S5(225)						***
S6(75)						

Significance is indicated at three probability levels

* p < 0.05

** p < 0.01

*** p < 0.001.

**Table 3:** Statistical comparison of mean collagen anisotropy at different depths for the inferior-temporal peripapillary scleral quadrant (two-tailed t-tests).

Section (mean depth, μm)	S1 (825)	S2 (675)	S3 (525)	S4 (375)	S5 (225)	S6 (75)
S1(825)		***	***	**		***
S2(675)					***	***
S3(525)					***	***
S4(375)					**	***
S5(225)						***
S6(75)						

Significance is indicated at three probability levels

* p < 0.05

** p < 0.01

*** p < 0.001.

**Table 4:** Statistical comparison of mean collagen anisotropy at different depths for the inferior-nasal peripapillary scleral quadrant (two-tailed t-tests).

Section (mean depth, μm)	S1 (825)	S2 (675)	S3 (525)	S4 (375)	S5 (225)	S6 (75)
S1(825)		**	***	***		***
S2(675)			***	***		***
S3(525)					***	***
S4(375)					***	***
S5(225)						***
S6(75)						

Significance is indicated at three probability levels

* p < 0.05

** p < 0.01

*** p < 0.001.

## Discussion

This paper presents, to the best of our knowledge, the first quantitative maps of collagen fibril organisation in the human peripapillary sclera at multiple tissue depths. We have documented significant depth-dependent changes in preferred fibril orientation and anisotropy that varied in their profile between different scleral quadrants. There were two main findings. Firstly, the previously described circumferential collagen ringing the nerve head was not continuous through the whole stromal depth, being largely restricted to the outer two-thirds of the tissue. In the contrast, the innermost layer of the scleral tissue was characterised by radially aligned collagen, possibly relating to the converging pattern of vessels when approaching the nerve head in the adjacent choroid layer. Secondly, the degree of collagen anisotropy was maximal in the mid-stroma and decreased when approaching the tissue surfaces, with marked region-specific variation. These observations are consistent with our prior multiphoton work[7] in which a semi-quantitative analysis of collagen anisotropy from SHG images at compatible depths displayed largely similar trends, albeit that previous study sampled much more limited, localised regions of the peripapillary sclera. A more recent study by Danford and co-workers[15] examined the depth-dependency of posterior scleral collagen orientation more quantitatively using SALS. In that study the authors measured a parameter (eccentricity) which is closely-related to our current anisotropy values and that exhibited a variation with depth consistent with the current results (i.e. reaching a maximum in the mid-stroma) in the superior and inferior scleral regions. However, in contrast to the present study, this trend was observed to alter in the nasal and temporal regions where eccentricity peaked in the penultimate stromal layer to the episclera. Moreover, the Danford et al study also reported that region-specific differences in eccentricity at the extraocular surface converged on approaching the mid-stroma and thereafter remained similar, whereas, in contrast, the current results indicated that region-specific differences in anisotropy existed across the whole stromal thickness. Inconsistencies between our work and that of Danford et al are likely due to two factors. Firstly, the subdivision of regions in their study (superior, inferior, nasal, temporal) differed from ours. Secondly, and more importantly, the Danford work used posterior scleral specimens removed at an averaged distance of 2.25mm from the canal edge, meaning that the great majority of their examined tissue was located outside the immediate peripapillary zone in which we found highly ordered, circumferentially arranged collagen. Our previous studies[5, 7] included data recorded from the mid-posterior scleral region also examined by Danford et al, and the results did not indicate the presence of significant circumferentially-aligned collagen. Taken together, the present work and that by Danford et al suggest that depth-related changes in posterior scleral structure likely also vary markedly as a function of their distance from the nerve head.

**Fig 7:**
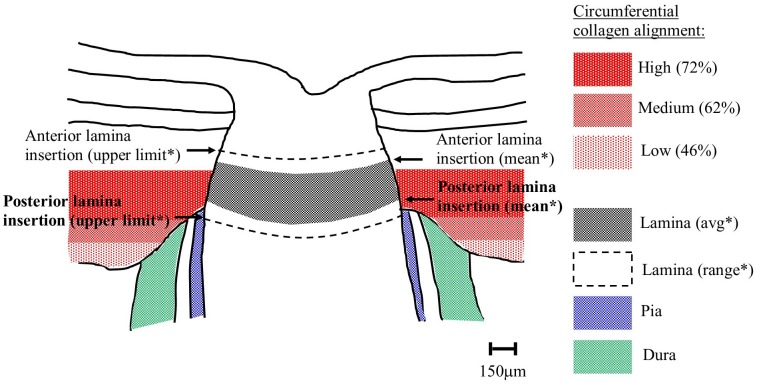
Comparison of circumferential collagen and lamina insertion depths. Schematic of human optic nerve and peripapillary sclera cross-section, showing the location of the circumferential scleral collagen, as determined by WAXS, in relation to the typical position of the lamina cribrosa in a generic middle-aged/elderly normal human eye. % circumferential alignment is expressed as mean anisotropy, averaged over the four quadrants of eight eyes. *Distances of lamina insertion sites (average and range) are taken from the literature[20–22, 26].

**Fig 8:**
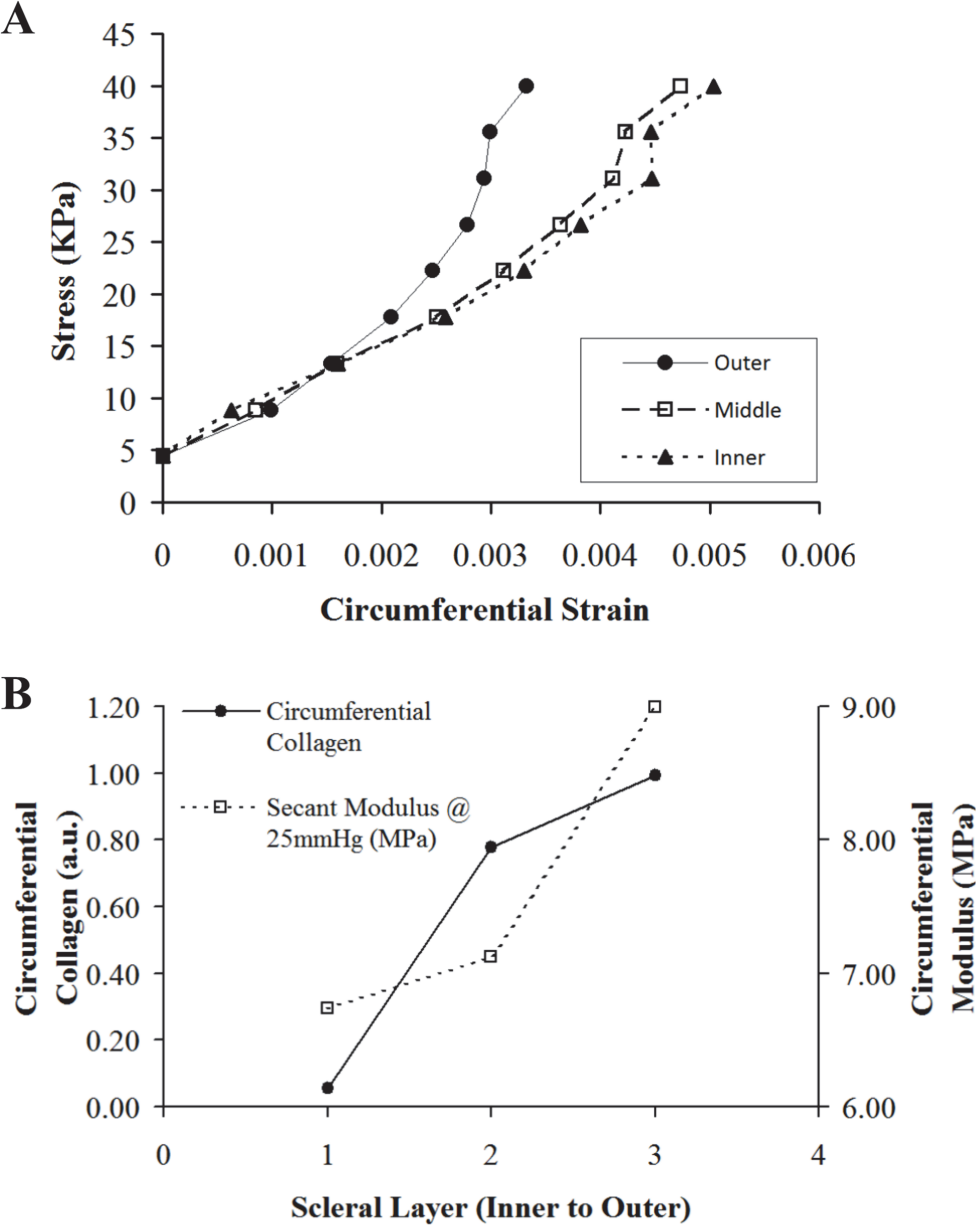
Depth-profile comparison of scleral mechanical behaviour and collagen microstructure. A) Comparison of circumferential stress-strain behaviour for inner, middle and outer layers of the human sclera, at an average distance of 2mm from the canal edge, based on data from previous ultrasound speckle tracking experiments[27]. B) Average circumferential secant modulus (tissue stiffness) at 25mmHg, calculated from data shown in A), compared with average x-ray scatter from circumferentially-aligned collagen (calculated from the current data).

Depth-dependent variations in the structure of the peripapillary sclera are of interest in the study of glaucoma, since this tissue region is thought to have some involvement in the development of the disease. An overall circumferential arrangement of collagen in the peripapillary sclera appears optimal for limiting canal expansion[11–13], and hence, presumably, represents a feature that would reduce strain in the lamina—a principal site of axonal damage in glaucoma[23]. Moreover, the mechanical properties[24] and extracellular matrix architecture[7, 10, 25] of the peripapillary sclera are altered in glaucomatous human eyes. We suspect that the depth-dependent changes in non-glaucoma eyes identified herein may also reflect a mechanical adaption of the connective tissue, specifically relating to the anatomical position of the lamina cribrosa with respect to the sclera. In normal human eyes the anterior lamina insertion sites into the scleral canal are located, on average, approximately 200μm from the intraocular scleral canal opening and 50μm from the posterior opening [20–22, 26] (see Fig. 7). Moreover, in older subjects, the posterior insertion frequently extends beyond the outer scleral canal boundary into the pia mater[21, 22, 26] (Fig. 7). This may explain our current observation that the peripapillary circumferential collagen structure is limited to the outer two-thirds of the stroma, with the highest circumferential alignment (anisotropy) being mid-stroma where the lamina is in direct contact with the sclera (Fig. 7). Significant regional variation in the site of lamina insertion, as reported elsewhere[22], may also link with the large differences in collagen anisotropy with location around the ONH noted in the current study (Figs. 5 and 6). Furthermore, in light of the present results, it is interesting that a recent study by Liu et al.[27] using ultrasound speckle-tracking[28, 29] reported that tangential (in-wall) strains in the human posterior sclera vary significantly through the stromal thickness, with averaged meridional and circumferential strains decreasing in the outer layers. In order to determine how these observations may relate to depth-related changes in tissue microstructure identified herein we calculated from the Liu et al.[27] study the circumferential stress-strain behaviour for the inner, middle and outer layers of the human posterior sclera (Fig. 8A). The in-wall stresses were estimated from the Laplace law using average scleral radius of curvature and thickness. Given that the average thickness of the peripapillary sclera was about 0.9 mm and considering potential variations of stresses from inner to outer sclera, a finite element model with the geometry of sclera was used to investigate the stress differences in the inner, middle, and outer thirds of the wall. Our results showed minimal deviations (with a maximum 2–3% error) in the estimate of wall stress using Laplace law. In addition, the wall stress also had minimal dependence on modulus. Subsequently, we used these stress values to calculate the average circumferential stiffness (i.e. secant modulus at a given stress) as a function of tissue depth (Fig. 8B). We then compared these modulus values (calculated at a pressure of 25mmHg) with the relative scatter from circumferentially-aligned collagen, calculated within the current specimens at a radius of 1.5–2.5mm from the canal edge (corresponding to the location of strain measurements in the Liu et al.[27] study) (Fig. 8B). The results are generally compatible with our hypothesis of increased circumferential collagen leading to elevated circumferential stiffness (reduced strains), and thus enhanced lamina support, in the mid/outer layers of the posterior sclera. However further mechanical evaluation of the immediate peripapillary tissue, ideally with finer depth resolution, is required to more effectively match to the present microstructural data. Moreover the presently unknown effects of inter-layer shear on the bulk behaviour of the sclera warrant investigation, ideally via a 3D, through-thickness mechanical evaluation of the peripapillary sclera.

This study was subject to a number of experimental limitations. Firstly, the collagen molecules from which the WAXS signal derives[19] are assembled into an intermediate microfibril structure with an inclination angle of ∼5°[30], which in turn are packed into fibrils. This has the effect of broadening the WAXS peaks, affecting the measured fibril dispersion. The consequence of this would be a minor under-estimation of our measured collagen anisotropy values, affecting all specimens equally. Secondly, sectioning of the tissue would have led to some mechanical disturbance at the specimen surfaces, affecting the local collagen architecture. Since WAXS averaged the full 150μm of the section thickness this artifact would likely have made a minor contribution. Thirdly, although the eyes used in the study all came from ostensibly normal donors (i.e. no history of scleral surgery or glaucoma) the scleral dimensions of the donors were not measured. It is not known if and how eye size is related to the collagen fibril orientation in the sclera. However, the posterior scleral matrix is well known to remodel at the level of the collagen fibril level during axial myopia[31, 32]. Since it is likely that the specimens presented here came from donors with a diversity of axial lengths, this may have contributed some variation in the measured collagen structure between eyes. Fourthly, flattening of the natural scleral curvature for x-ray examination may have released some of the residual stress present within the intact tissue, potentially leading to changes in the natural collagen orientation. However, studies in other collagenous tissues suggest that this effect tends to be more evident at the macro (organ) level and less so at the level of the collagen microstructure[33]. Lastly, all the eyes studied came from donors aged 53 years and above. Given that anatomical and other changes in the posterior eye occur with age, for example thickening of the lamina cribrosa[34], it is possible that the results presented in the current study may not be representative of younger eyes, but are more characteristic of the typical age for glaucoma. Furthermore, given the range of donor ages (53–79 yrs) present in the present samples, possible effects of age on the measured collagen microstructure cannot be ruled out.

In summary, this study has shown that significant, region-specific variations in collagen structure are present in the human peripapillary sclera as a function of depth. In normal eyes, the predominantly circumferential collagen fibril architecture is most prominent in the outer two-thirds of the stromal thickness. This arrangement would logically be consistent with more effective support of the lamina cribrosa at the level of its insertion into the scleral canal wall. Further study of glaucomatous changes in the mechanical and microstructural properties of the peripapillary sclera as a function of depth are warranted, particularly as recent research suggests that depth-dependent collagen orientation in the posterior tissue is altered in human glaucoma eyes[15]. Depth-profiled, quantitative data on scleral collagen orientation, such as presented here, will benefit computational modelling efforts aimed at characterising the biomechanical behaviour of the sclera and ONH, and their potential role in glaucoma.
